# Racial and ethnic disparities in access to acute stroke capabilities in California: Association with rurality and telestroke access

**DOI:** 10.1016/j.jstrokecerebrovasdis.2026.108607

**Published:** 2026-03-10

**Authors:** Kori S. Zachrison, Renee Y. Hsia, Krislyn M. Boggs, Jingya Gao, Luke Messac, Lee H. Schwamm, Mathew J. Reeves, Vicki Fung, Margaret E. Samuels-Kalow, Carlos A. Camargo

**Affiliations:** a Department of Emergency Medicine, Massachusetts General Hospital, Boston, MA, USA; b Harvard Medical School, Boston, MA, USA; c Department of Emergency Medicine, University of California San Francisco, San Francisco, CA, USA; d Department of Emergency Medicine, Brigham & Women’s Hospital, Boston, MA, USA; e Departments of Neurology and Biomedical Informatics & Data Sciences, Yale School of Medicine, New Haven, CT, USA; f Department of Epidemiology and Biostatistics, Michigan State University, East Lansing, MI, USA; g Mongan Institute Health Policy Research Center, Massachusetts General Hospital, Boston, MA, USA

**Keywords:** Telestroke, Acute stroke, Access to care, Rural disparities, Racial/ethnic disparities

## Abstract

**Background:**

We sought to examine the relationship between patient race/ethnicity and presentation to an emergency department (ED) with acute stroke capabilities, and whether this varied by rurality and telestroke access.

**Methods:**

All acute ischemic stroke encounters in California in 2021 were obtained from California Department of Health Care Access and Information data. ED capabilities were from the 2021 National ED Inventory-USA database. Acute stroke capability was defined as having acute stroke ready status (or higher), telestroke or both. We examined the association between race/ethnicity and presentation to an ED with acute stroke capabilities, overall and stratified by rurality. Sensitivity analyses removed telestroke from our definition of acute stroke capability.

**Results:**

In 2021, 264 of 325 California EDs (81%) had acute stroke capabilities, 41 (13%) via telestroke alone. Only 2,050 of 63,252 encounters (3%) presented to an ED without capabilities. Rural (versus urban) patients had lower odds of access to acute stroke capabilities, regardless of telestroke status. There were no differences in access by race/ethnicity for rural patients, but urban Hispanic, non-Hispanic Black and patients of other race had lower odds of access (versus urban non-Hispanic Whites) regardless of telestroke status.

**Conclusions:**

Most California EDs have acute stroke capabilities. While few patients presented to non-capable centers, rural patients had lower odds of access. Racial/ethnic disparities differed between urban and rural settings, with lower odds of access for Hispanic and non-White urban patients but no significant differences by race/ethnicity in rural patients, however this latter finding might reflect limited power due to smaller sample sizes. ED telestroke capability did not reduce disparities in access.

## Background

Stroke centers have dedicated resources for the management of patients with stroke and deliver higher quality of care for patients with acute ischemic stroke.^1^ Yet most US emergency departments (EDs) are not in stroke center hospitals, and some patients lack immediate access to stroke center care.^2,3^ There are also important geographic and demographic disparities in access, with longer distances to stroke centers for patients from rural areas and from census tracts with more older, American Indian, uninsured, and low-income individuals.^4^ The impact of rurality may also vary by race or ethnicity; for example, Black patients in rural areas have been identified as a particularly high-risk group.^5^ Differences in access are important contributors to known disparities in care delivery and outcomes for rural patients with stroke.^6,7^

For patients with acute stroke presenting to EDs with limited access to on-site neurological expertise, telestroke is a guideline-recommended strategy for bringing remote expertise to the bedside and is associated with improved care and outcomes.^8–10^ Some hospitals that would not otherwise have 24/7 neurology access also use telestroke to supplement coverage and obtain stroke center certification. It is possible that telestroke capacity may mitigate racial/ethnic and rural disparities in access to stroke centers by providing specialty neurological care in EDs that would not otherwise have access. However, ED telehealth capability may vary by community payer mix.^11^ If EDs serving more medically underserved or racially/ethnically diverse communities are also less likely to have telestroke, then differences in telestroke capacity between EDs could further exacerbate inequities in access, care and outcomes.^6,7^ This relationship may also vary by geography; for example, there may be distinct patterns in a state such as California, where a higher proportion of the rural population is Hispanic.^12^

The association between telestroke and racial/ethnic disparities in access to acute stroke expertise has not been well-described. To address this knowledge gap, we examined the relationship between patient race/ethnicity and odds of presenting to an ED with acute stroke capabilities – whether defined by stroke center status or telestroke capacity, and whether this varied by rurality using statewide California data. Because disparities in access may also be impacted by structural determinants, we also examined whether there was an association between demographic and socioeconomic characteristics of the population living near an ED and its likelihood of having acute stroke capabilities.

## Methods

### Data sources and population

Our study setting was the state of California, where there is not a unified statewide approach to prehospital routing for suspected stroke. We used non-public data maintained by the California Department of Health Care Access and Information (HCAI). The data include all ED and hospital discharges from all non-federal, acute care hospitals licensed in California. We identified all encounters for acute ischemic stroke during the 2021 calendar year using primary diagnosis codes (International Classification of Disease, Tenth Revision [ICD-10-CM], and Medicare Severity Diagnosis Related Groups [MS-DRG]).^13^

We used ED-level data from the 2021 National ED Inventory (NEDI)-California and -USA surveys. The survey administration was coordinated by the Emergency Medicine Network, with methods previously reported.^14^ NEDI-USA includes all US EDs open 24/7 and available for use by the general public (both hospital-based and freestanding); the survey excludes EDs at federal hospitals and college infirmaries that are not necessarily available for use by the general public.

The NEDI-USA survey was administered in 2022 to characterize EDs in 2021. The survey includes questions on basic characteristics, staffing, and telemedicine use (Supplement). The telemedicine questions distinguish between provision and receipt, clinical indications for use, and include a distinct telestroke field.^15^ In California, we expanded the NEDI-USA survey to include additional questions to characterize EDs’ stroke-related capabilities and care patterns (NEDI-California; Supplement). Data were collected from February-December 2022. Surveys were completed on paper, online, or by telephone. We initially reached out to all California EDs with the expanded NEDI-California survey. The survey was mailed to ED directors up to 3 times over a 3-month period, including a link to the online version. If a NEDI-USA respondent from a prior year provided an email address, we emailed an electronic copy of the survey to this contact in place of the first paper mailing. Follow-up to nonresponsive sites and those with partially completed surveys was conducted by telephone. In November 2022, we reverted to the one-page NEDI-USA survey in follow-up calls to optimize data collection for the most key variables (e.g., telestroke capability). REDCap electronic data capture tool was used to manage survey data.

We used Social Vulnerability Index (SVI) to characterize the neighborhood surrounding an ED based on demographic and socioeconomic factors.^16^ This measure includes factors such as poverty and transportation access, and accounts for area racial/ethnic composition. We used the ED’s coordinates to link to its census tract and obtain SVI for the associated census tract. SVI was based on state percentile rankings as a range from 0 to 1 and included in our models as a continuous variable.

### Outcome of interest

Our primary outcome of interest was at the encounter level, based on whether a patient presented to an ED with acute stroke capabilities. We first defined an ED with acute stroke capabilities as one with stroke center status (equivalent to acute stroke ready hospital [ASRH] or higher) or telestroke capacity. To determine the effect of telestroke, we subsequently restricted the definition to exclude telestroke capacity. Stroke center status was identified using our previously developed inventory including all certifying bodies.^2^ Telestroke capacity was based on response to the telestroke question on the survey, as detailed above. If a site did not respond to the survey or to the telestroke question for 2021, then telestroke status from 2020 was used when available.

### Analysis and other covariates of interest

Patient encounters were categorized based on whether the presentation was to an ED with acute stroke capabilities (0/1) based on presentation to an ED with ASRH status or higher or telestroke (primary definition). Then, to examine the extent to which telestroke may mitigate disparities in access to an ED with acute stroke capabilities, we excluded telestroke from the definition. Characteristics of EDs with and without acute stroke capabilities (based on our primary definition) were compared using Wilcoxon rank sum and Chi-square tests as appropriate.

Our exposures of interest were patient race/ethnicity and rurality. Race/ethnicity is captured based on HCAI documentation and categorized as non-Hispanic White, non-Hispanic Black, Hispanic, non-Hispanic Asian or Pacific Islander, other, and not reported. Rurality was based on the rurality of the ED where the patient presented, using urban influence codes (UIC) from the Office of Management and Budget, defining rural as nonmetropolitan counties (codes 7–9).^17^

Other patient-level covariates were age, sex, and expected payer (private insurance, Medicare, Medicaid or uninsured). To characterize patients and their encounters and treatment course, we also descriptively examined: Charlson comorbidity index, receipt of thrombolytic, receipt of thrombectomy, and disposition from initial ED (discharge, transfer, or admission). Encounters missing race/ethnicity, sex, or payer information were excluded from the analysis (3%).

Other ED-level covariates based on HCAI or NEDI-USA data were total annual visit volume and total ischemic stroke volume in 2021, academic status, critical access hospital status, telestroke capacity and stroke center status (ASRH, primary stroke center [PSC], thrombectomy-capable stroke center [TSC], comprehensive stroke center [CSC]). ED-level covariates that were additionally available among EDs responding to the NEDI-California survey were neuroimaging availability (non-contrast CT, CT angiography, CT perfusion, MRI) and presence of a stroke protocol.

To examine the relationship between patient race/ethnicity, rurality and likelihood of presenting to an ED with acute stroke capabilities, we fit encounter-level multivariable logistic regression models. We then repeated the models stratified by rurality based on our a priori interest in understanding the potential interaction between rurality and race/ethnicity. The outcome of interest was initial presentation to an ED with acute stroke capabilities, based on our primary definition. The independent variables of interest were race/ethnicity and rurality. We first examined the unadjusted relationships, then adjusted for race/ethnicity and rurality together, next additionally included age, sex and expected payer to account for potential confounding effects of demographic characteristics.

We performed a sensitivity analysis to examine whether telestroke mitigated disparities in access based on stroke center status. To do this we excluded telestroke from the definition of acute stroke capability so that the outcome was based on presentation to an ED with ASRH status or higher only and repeated the above analyses.

At the ED level, we used a multivariable logistic regression model to examine whether there was an association between the SVI for the census tract where an ED is located and its likelihood of having acute stroke capabilities. The outcome of interest was presence of acute stroke capabilities as defined above. The independent variables of interest were ED census tract SVI and rurality. Other model covariates included in the model were included to take into account other characteristics related to an EDs’ level of resources, namely critical access hospital status, academic status, and annual stroke volume (no visits, <60, 60–120, >120, unknown).

## Results

### Patient characteristics

There were 63,252 patient encounters included in our final sample, only 2,050 (3%) of whom initially presented to an ED without acute stroke capabilities. Patients presenting to EDs without acute stroke capabilities were more often Non-Hispanic Black or Hispanic with Medicaid insurance or self-pay, presenting to a rural ED, and were more often transferred out from the initial ED (Table 1).

### ED characteristics

There were 325 (97%) California EDs included in our analysis (Supplemental Figure), of which 31 (10%) were rural and 264 (81%) had acute stroke capabilities through location in a hospital with ASRH status or higher (*n* = 223) or telestroke alone (*n* = 41). Of the 159 EDs in a hospital with ASRH or PSC status, 97 (61%) had telestroke. EDs without acute stroke capabilities had lower annual visit volume, were more often rural, more often critical access hospitals and had lower annual stroke volume (Table 2). Among the 41% of EDs completing the 2021 NEDI-California survey (*n* = 134), availability of non-contrast head CT was similar, but otherwise stroke-related resources were less frequent in EDs without acute stroke capabilities relative to those with capabilities (Table 2).

### Race/ethnicity, rurality and access to acute stroke capabilities

Overall, relative to non-Hispanic white patients, Hispanic patients were less likely to present to an ED with acute stroke capabilities in both unadjusted and adjusted models. The lower odds of presentation to an ED with acute stroke capabilities among Hispanic patients held whether telestroke was included in the definition of stroke capabilities or not (Table 3). After stratifying by rurality, the odds of presenting to an ED with acute stroke capabilities were not significantly different for rural Hispanic vs. non-Hispanic white patients. This was consistent in unadjusted and adjusted models. The absence of difference for rural Hispanic vs. non-Hispanic white patients was also consistent whether telestroke capability was included in the definition of stroke capabilities or not (Table 4). In contrast, urban Hispanic patients had lower odds of presenting to an ED with acute stroke capabilities in unadjusted and adjusted models compared with urban non-Hispanic white patients. The lower odds of presenting to an ED with acute stroke capabilities for urban Hispanic patients relative to urban non-Hispanic white patients was consistent regardless of whether telestroke was included in the definition for stroke capabilities or not (Table 4).

Relative to non-Hispanic white patients, in unadjusted analysis non-Hispanic Black patients had similar odds of presenting to an ED with acute stroke capabilities but lower odds in all adjusted analyses (Table 3). When stratified, the odds of presenting to an ED with acute stroke capabilities did not differ significantly for rural non-Hispanic Black vs. white patients, whereas, urban non-Hispanic Black patients had lower odds in unadjusted and adjusted analyses regardless of whether telestroke capability was included in the definition (Table 4). Non-Hispanic patients of other race followed the same trend as non-Hispanic Black patients.

### Characteristics of ED location and likelihood of acute stroke capabilities

There was no association between the neighborhood SVI of the census tract where the of ED was located and its likelihood of having acute stroke capabilities. This was true in bivariate analysis as well as after adjusting for other characteristics of the ED (Supplemental Table 1). The only ED characteristic that was associated with having acute stroke capabilities was an ED’s annual stroke volume, with acute stroke capabilities being less likely among lower volume EDs.

## Discussion

In this comprehensive statewide analysis, we found that relative to non-Hispanic White patients, Hispanic and non-Hispanic patients of other races were less likely to present to EDs with acute stroke capabilities. Excluding EDs with telestroke capabilities did not change this finding, indicating that telestroke did not mitigate this racial/ethnic disparity. The racial/ethnic disparity was pronounced in urban settings, where Hispanic patients, non-Hispanic Black patients and non-Hispanic patients of other races all had lower odds of presenting to an ED with acute stroke capabilities, regardless of telestroke capabilities. In contrast, rural patients had lower odds of presenting to an ED with acute stroke capabilities across the board. While we found no significant variation by race/ethnicity in the rural setting, this may have been related to smaller sample size.

We hypothesized that racial/ethnic and rural differences in access may be due to lower acute stroke capabilities among EDs serving communities with more social vulnerability. However, we did not find any association between the SVI surrounding an ED and its acute stroke capabilities. This finding is in contrast to other literature demonstrating that hospitals serving low-income and rural communities have been less likely to adopt stroke center certification^18^ and less likely to have telehealth in their EDs.^11^ There are many potential explanations for the difference in findings. For example, the SVI of an ED’s census tract may not reflect its full catchment area (i.e., misclassification of the exposure). Differences in measurements used to characterize social vulnerability of a community may also have contributed to different findings. This may also be related to hospital-level financial variables. For example, Black-serving hospitals have historically lower reimbursements – even when not in impoverished census tracts – and thus may have fewer resources to invest in stroke-related capabilities.^19^ Other potential explanations for the difference between our findings and other work include differences in population studies (national versus statewide data) or differences in the outcome of interest. We tested whether this finding was due to our definition of acute stroke capabilities and found that excluding telestroke from the definition did not impact our results.

We also found lower odds of presentation to an ED with acute stroke capabilities for patients with Medicaid or self-pay insurance. This finding, as well as the patient-level finding of rural and racial/ethnic disparities in ED presentation is similar to other studies that have repeatedly demonstrated that access to reperfusion therapy and to stroke center care is lower for vulnerable populations.^20–27^ However, less research has examined access via telestroke, which has been promoted as a tool for improving access in under-resourced settings. A study from Texas, a state with a sizeable rural population, found that telestroke expansion was not associated with changes in racial/ethnic disparities in access for the state and that, in fact, there were not racial or ethnic disparities in access to stroke center or telestroke-equipped hospitals.^28^ Unfortunately, we found that in California the disparity in access persists even with telestroke.

In this context, it is critical to consider how to address these persistent access disparities. One possible solution is to improve the medical services where patients initially present. Among patients transported by emergency medical services (EMS), ensuring robust stroke routing protocols is critical. Yet many patients do not arrive by EMS, and even among those who do, if a community lacks a nearby hospital with adequate resources or expertise, a routing protocol will not address the issue. Additional considerations include improving interhospital transfer systems to move patients to the appropriate level of resources, and to overcome existing inequities in transfer processes^27,29^ by developing coordinated regional transfer systems.

Our study has important limitations. The California-NEDI survey response rate was lower than anticipated; as a result of the low response rate and differences between respondents and non-respondents (Supplemental Table 2), we were unable to examine differences in access to other components of ED-based stroke resources. However, we were able to examine the variable of greatest interest, namely telestroke. Even so, we captured telestroke use dichotomously and did not have details on the nature of use (e.g., for particular hours or subsets of patients), and thus may have misclassified use. Other concerns related to misclassification of EDs are related to the reported stroke-related capabilities of EDs presented in Table 2, where we found that not all EDs with stroke certification or telestroke reported 24/7 access to non-contrast CT or thrombolytic. Whether this represents misclassification or documentation errors related to CT capabilities requires further study. A second important note is that we defined rurality based on the zip code of the ED where a patient presented rather than the zip code of the patient, thus some rural residents may not have been categorized as rural if they presented or were transported to an urban ED. While this may have underestimated rural resident representation in the data, it does capture patterns of presentation and the impact of receiving care in a rural ED. Additionally, at the encounter level we had limited measures of patient acuity or need for a higher level of resources in this administrative data, and thus were unable to account for these factors that could confound associations. We also lacked data on mode of arrival (i. e., EMS vs. self-transport) which limits our ability to understand differences in decision-making related to ED destination choice. It is also important to note that this study may be underpowered to detect racial/ethnic differences in the rural setting. Relatedly, we did not specifically examine patients identifying as American Indian or Alaska Native. While this is a sizeable population in California, the sample size in our study (*n* = 6,872, with *n* = 19 rural) was not sufficient to support a distinct subgroup analysis. Our study was thus limited in the ability to assess potential disparities in access for this historically underserved population. Finally, this is a statewide analysis with high penetrance of stroke-capable EDs and results may not be generalizable to other settings with different healthcare market, population and geographic characteristics. Even so, California is a large state, with over 10% of the US population, and understanding the stroke system of care in the state has the potential to inform improved organization and access for millions of patients.

## Conclusion

In a comprehensive statewide analysis, we found that most California EDs have acute stroke capabilities. While few patients presented to non-capable centers, rural patients had lower odds of access. Racial/ethnic disparities differed between urban and rural settings, with no significant difference detected by race/ethnicity among rural patients but lower odds of access for Hispanic and non-White urban patients. ED telestroke capability did not reduce disparities in access.

## Supplementary Material

1

Supplementary material associated with this article can be found, in the online version, at doi:10.1016/j.jstrokecerebrovasdis.2026.108607.

## Figures and Tables

**Table 1:** Patient characteristics, stratified by initial presentation to ED with acute stroke capabilities*.

Patient Characteristics	Overall Sample	Presenting to EDs with acute stroke capabilities*	Presenting to EDs without acute stroke capabilities	p-value
	*n* = 63,252	*n* = 61,202	*n* = 2,050	

Age, median (IQR)	71 (61–81)	72 (61–81)	68 (58–78)	<0.001
Female, n (%)	30,280 (48)	29,378 (48)	902 (44)	<0.001
Race/ethnicity, n (%)				<0.001
Non-Hispanic White	30,990 (49)	30,161 (49)	829 (40)	
Non-Hispanic Black	6,868 (11)	6,611 (11)	257 (13)	
Hispanic	14,644 (23)	14,030 (23)	614 (30)	
Non-Hispanic other^^^	10,750 (17)	10,400 (17)	350 (17)	
Expected payer, n (%)				<0.001
Private	11,117 (18)	10,810 (18)	307 (15)	
Medicare	41,076 (65)	39,954 (65)	1,122 (55)	
Medicaid	10,230 (16)	9,646 (16)	584 (28)	
Self-pay	829 (1)	792 (1)	37 (2)	
Charlson comorbidity index, median (IQR)	4 (2–6)	4 (2–6)	3 (2–5)	<0.001
Receipt of thrombolytic, n (%)	5,897 (9)	5,846 (10)	51 (2)	<0.001
Receipt of thrombectomy, n (%)	2,494 (4)	2,489 (4)	<11 (<1)^^^^	<0.001
Presentation to rural ED, n (%)	1,446 (2)	1,157 (2)	289 (14)	<0.001
Disposition, n (%)				<0.001
Discharge	9,128 (14)	8,861 (14)	267 (13)	
Transfer	3,816 (6)	3,473 (6)	343 (17)	
Admission	49,485 (78)	48,088 (79)	1,397 (68)	
Other/Missing	823 (1)	780 (1)	43 (2)	

ED emergency department; IQR interquartile range.

* Acute stroke capabilities defined as presence of telestroke or in an acute stroke ready hospital, primary, thrombectomy-capable, or comprehensive stroke center.

^ This group includes 6,872 American Indian/Alaska Natives.

^^ Values masked due to small cell size.

**Table 2:** ED Characteristics, stratified by acute stroke capabilities*.

ED Characteristics	Overall Sample	EDs with acute stroke capabilities*	EDs without acute stroke capabilities	p-value for difference

	*n* = 325	*n* = 264	*n* = 61	
*Full ED sample (n = 325*)^^^				
2021 visit volume Median (IQR)	38,031 (20,008–60,000)	42,942 (25,135–63,938)	17,450 (7,711–33,402)	<0.001
Rural location, n (%)	31 (10)	17 (6)	14 (23)	<0.001
CAH status, n (%)	36 (11)	19 (7)	17 (28)	<0.001
Social vulnerability index where ED is located, median (IQR)	0.59 (0.37–0.80)	0.56 (0.36–0.79)	0.68 (0.46–0.84)	0.04
Academic, n (%)	21 (6)	19 (7)	2 (3)	0.39
Total ischemic stroke volume in 2021, n (%)				<0.001
None	5 (2)	0 (0)	5 (8)	
< 60 cases	77 (24)	38 (14)	39 (64)	
60–120	53 (16)	46 (17)	7 (11)	
121–240	57 (18)	53 (20)	4 (7)	
>240	115 (36)	114 (43)	1 (2)	
Unknown	18 (5)	13 (5)	5 (8)	
Telestroke capability, n (%)	167 (51)	167 (63)	0 (0)	n/a
Stroke center status, n (%)				n/a
None	102 (31)	41(16)	61 (100)	
ASRH	12 (4)	12 (5)	0 (0)	
PSC	147 (45)	147 (56)	0 (0)	
TSC	22(7)	22 (8)	0 (0)	
CSC	42 (13)	42 (16)	0 (0)	
*NEDI-California respondents (n = 134*)^^^^				
Respondents, n (%)	134 (41)	104 (39)	30 (49)	
Stroke-related resource availability				
Non-contrast CT, n (%)	107 (80)	82 (79)	25 (83)	0.24
CT angiography of head, n (%)	104 (78)	81 (78)	23 (77)	0.04
CT perfusion, n (%)	70 (52)	57 (55)	13 (43)	0.04
MRI, n (%)	91 (68)	74 (71)	17 (57)	0.001
Thrombolytic available, n (%)	98 (73)	77 (74)	21 (70)	0.06
Stroke protocol, n (%)	107 (80)	84 (81)	23 (77)	0.01

CAH: critical access hospital.

* Acute stroke capabilities defined as presence of telestroke or in an acute stroke ready hospital, primary, thrombectomy-capable, or comprehensive stroke center.

^ Among the full ED sample, ED characteristics based on HCAI or NEDI-USA data.

^^ Among EDs responding to the NEDI-California survey, ED characteristics based on NEDI-California.

**Table 3:** Relationship between patient race/ethnicity, rurality and access to acute stroke capabilities.

	Sample size n (%)	Outcome: Access to ED with ASRH equivalent or higher or telestroke capability*			Outcome: Access to ED with ASRH equivalent or higher^^^		
	
		Unadjusted OR (95% CI)	OR adjusted for race/ethnicity and rurality (95% CI)	OR additionally adjusted for age, sex and insurance (95% CI)	Unadjusted OR (95% CI)	OR adjusted for race/ethnicity and rurality (95% CI)	OR additionally adjusted for age, sex and insurance (95% CI)

Race/ethnicity							
NH-white	30,990 (49)	Reference	Reference	Reference	Reference	Reference	Reference
Hispanic	6,868 (11)	0.63 (0.560.70)	0.48 (0.42–0.53)	0.54 (0.48–0.61)	0.74 (0.690.79)	0.54 (0.50–0.59)	0.60 (0.56–0.65)
NH-black	14,644 (23)	0.71 (0.610.82)	0.53 (0.45–0.61)	0.60 (0.51–0.69)	1.02 (0.921.13)	0.74 (0.67–0.83)	0.82 (0.74–0.92)
NH-other	10,750 (17)	0.82 (0.720.93)	0.65 (0.57–0.74)	0.68 (0.60–0.78)	0.93 (0.851.01)	0.72 (0.66–0.79)	0.74 (0.68–0.81)
Rural	1,446 (2)	0.12 (0.100.13)	0.09 (0.08–0.10)	0.09 (0.08–0.10)	0.07 (0.060.08)	0.05 (0.05–0.06)	0.06 (0.05–0.06)
Urban	61,806 (98)	Reference	Reference	Reference	Reference	Reference	Reference
Age							
<45	2,960 (5)	Reference		Reference	Reference		Reference
45–64	17,734 (28)	1.24 (1.031.49)		1.18 (0.98–1.43)	1.15 (1.011.31)		1.14 (0.99–1.31)
65–85	32,785 (52)	1.74 (1.462.08)		1.15 (0.93–1.43)	1.42 (1.241.61)		1.18 (1.01–1.38)
86+	9,773 (15)	2.03 (1.652.51)		1.22 (0.95–1.56)	1.88 (1.612.18)		1.48 (1.24–1.76)
Female	30,280 (48)	1.17 (1.081.28)		1.12 (1.02–1.22)	1.13 (1.061.20)		1.08 (1.01–1.15)
Male	32,972 (52)	Reference		Reference	Reference		Reference
Insurance							
Private	11,117 (18)	Reference		Reference	Reference		Reference
Medicare	41,076 (65)	1.01 (0.891.15)		1.02 (0.87–1.19)	0.93 (0.851.01)		0.90 (0.81–1.01)
Medicaid	10,230 (16)	0.47 (0.410.54)		0.53 (0.45–0.61)	0.54 (0.490.60)		0.59 (0.53–0.65)
Self-pay	829 (1)	0.61 (0.430.86)		0.65 (0.46–0.93)	0.65 (0.500.83)		0.68 (0.53–0.87)

ED emergency department, ASRH acute stroke ready hospital, OR odds ratio, CI confidence interval, NH non-Hispanic.

* The dependent variable for these encounter level models was presentation to an ED with ASRH status or higher or telestroke capability (*n* = 264) versus an ED without acute stroke capabilities (*n* = 61).

^ The dependent variable for these encounter level models was presentation to an ED with ASRH status or higher (*n* = 263) versus an ED without ASRH status or higher (*n* = 102).

**Table 4:** Relationship between patient race/ethnicity and access to acute stroke capabilities, stratified by rurality.

			Access to ED with ASRH or higher or telestroke		Access to ED with ASRH or higher	
		Sample size n (%)	Unadjusted OR (95% CI)	Adjusted for age, sex and insurance (95% CI)	Unadjusted OR (95% CI)	Adjusted for age, sex and insurance (95% CI)

RURAL (*N* = 1,446)	Race/ethnicity					
	NH-white	1,276 (88)	Reference	Reference	Reference	Reference
	Hispanic	57 (4)	0.76 (0.41–1.41)	0.86 (0.45–1.63)	1.45 (0.84–2.48)	1.55 (0.89–2.69)
	NH-black	12 (1)	2.72 (0.35–21.17)	2.16 (0.28–16.88)	0.49 (0.15–1.63)	0.48 (0.14–1.61)
	NH-other	101 (7)	0.94 (0.57–1.55)	0.93 (0.56–1.55)	0.67 (0.44–1.01)	0.70 (0.46–1.07)
	Age category					
	<45	36 (2)	Reference	Reference	Reference	Reference
	45–64	376 (26)	1.88 (0.90–3.92)	1.76 (0.81–3.82)	2.04 (0.98–4.27)	2.13 (1.004–4.52)
	65–85	824 (57)	2.16 (1.06–4.41)	1.74 (0.74–4.10)	2.34 (1.14–4.82)	2.98 (1.32–6.74)
	86+	210 (15)	1.94 (0.90–4.19)	1.55 (0.63–3.83)	2.86 (1.34–6.11)	3.57 (1.53–8.34)
	Female	682 (47)	1.31 (1.01–1.70)	1.24 (0.95–1.62)	1.11 (0.90–1.37)	1.09 (0.89–1.35)
	Male	764 (53)	Reference	Reference	Reference	Reference
	Insurance					
	Private	156 (11)	Reference	Reference	Reference	Reference
	Medicare	1,050 (73)	2.02 (1.39–2.93)	1.91 (1.18–3.08)	1.09 (0.78–1.52)	0.83 (0.54–1.26)
	Medicaid	224 (15)	2.31 (1.42–3.77)	2.32 (1.40–3.83)	1.03 (0.69–1.56)	1.20 (0.78–1.83)
	Self-pay	16 (1)	0.36 (0.13–1.01)	0.39 (0.14–1.13)	0.35 (0.11–1.14)	0.37 (0.11–1.21)
URBAN (*N* = 61,806)	Race/ethnicity					
	NH-white	29,714 (48)	Reference	Reference	Reference	Reference
	Hispanic	14,587 (24)	0.46 (0.41–0.52)	0.53 (0.47–0.60)	0.53 (0.49–0.58)	0.60 (0.55–0.64)
	NH-black	6,856 (11)	0.51 (0.44–0.59)	0.59 (0.50–0.68)	0.74 (0.66–0.83)	0.82 (0.74–0.92)
	NH-other	10,649 (17)	0.62 (0.54–0.71)	0.65 (0.57–0.75)	0.72 (0.65–0.78)	0.74 (0.68–0.81)
	Age category					
	<45	2,924 (5)	Reference	Reference	Reference	Reference
	45–64	17,358 (28)	1.27 (1.05–1.53)	1.14 (0.94–1.38)	1.20 (1.04–1.38)	1.11 (0.97–1.28)
	65–85	31,961 (52)	1.89 (1.57–2.28)	1.13 (0.91–1.42)	1.56 (1.36–1.78)	1.13 (0.96–1.32)
	86+	9,563 (15)	2.25 (1.80–2.81)	1.24 (0.96–1.61)	2.06 (1.76–2.42)	1.42 (1.18–1.70)
	Female	29,598 (48)	1.16 (1.05–1.27)	1.10 (0.995–1.21)	1.14 (1.06–1.21)	1.08 (1.01–1.15)
	Male	32,208 (52)	Reference	Reference	Reference	Reference
	Insurance					
	Private	10,961 (18)	Reference	Reference	Reference	Reference
	Medicare	40,026 (65)	1.02 (0.88–1.17)	0.94 (0.79–1.11)	1.01 (0.92–1.10)	0.91 (0.81–1.02)
	Medicaid	10,006 (16)	0.42 (0.36–0.48)	0.46 (0.39–0.53)	0.53 (0.48–0.59)	0.57 (0.51–0.63)
	Self-pay	813 (1)	0.68 (0.45–1.01)	0.71 (0.48–1.05)	0.68 (0.52–0.88)	0.70 (0.54–0.92)

ED emergency department, ASRH acute stroke ready hospital, OR odds ratio, CI confidence interval, NH non-Hispanic.
