# Supplementary material for: Racial and ethnic disparities in access to acute stroke capabilities in California: Association with rurality and telestroke access
Source: J Stroke Cerebrovasc Dis. Author manuscript; Available in PMC 2026 Apr 1. (PMC13043215; doi:10.1016/j.jstrokecerebrovasdis.2026.108607)
Supplement: 1 [file NIHMS2158450-supplement-1.docx]

**SUPPLEMENT**

Persistent Rural and Racial and Ethnic Disparities in Access to Acute Stroke Capabilities in California, Even with Telestroke

Kori S. Zachrison MD, MSc^1,2^, Renee Hsia MD, MS^3^, Krislyn M. Boggs MPH^1^, Jingya Gao MS^1^, Luke Messac MD, PhD^2,4^, Lee H. Schwamm MD^5^, Mathew J. Reeves BVSc, PhD^6^, Vicki Fung PhD^2,7^, Margaret E. Samuels-Kalow MD, MPhil, MSPH^1,2^, Carlos A. Camargo, Jr MD, DrPH^1,2^

^1^Department of Emergency Medicine, Massachusetts General Hospital, Boston, MA

^2^Harvard Medical School, Boston, MA

^3^Department of Emergency Medicine, University of California San Francisco, San Francisco, CA

^4^Department of Emergency Medicine, Brigham & Women’s Hospital, Boston, MA

^5^Departments of Neurology and Biomedical Informatics & Data Sciences, Yale School of Medicine, New Haven, CT

^6^Department of Epidemiology and Biostatistics, Michigan State University, East Lansing, MI

^7^Mongan Institute Health Policy Research Center, Massachusetts General Hospital, Boston, MA

Contact Information:

Kori S. Zachrison, MD, MSc

[kzachrison@mgh.harvard.edu](mailto:kzachrison@mgh.harvard.edu)

Table of Contents:

1. NEDI-USA Survey
2. NEDI-California Survey
3. Supplemental Figure. Flowchart of California Emergency Departments Included
4. Supplemental Table 1. Relationship between Hospital Characteristics and ED Stroke Capabilities
5. Supplemental Table 2. Characteristics of Respondent vs. Non-Respondent EDs to the NEDI-California Survey
6. 2021 NEDI-USA Survey


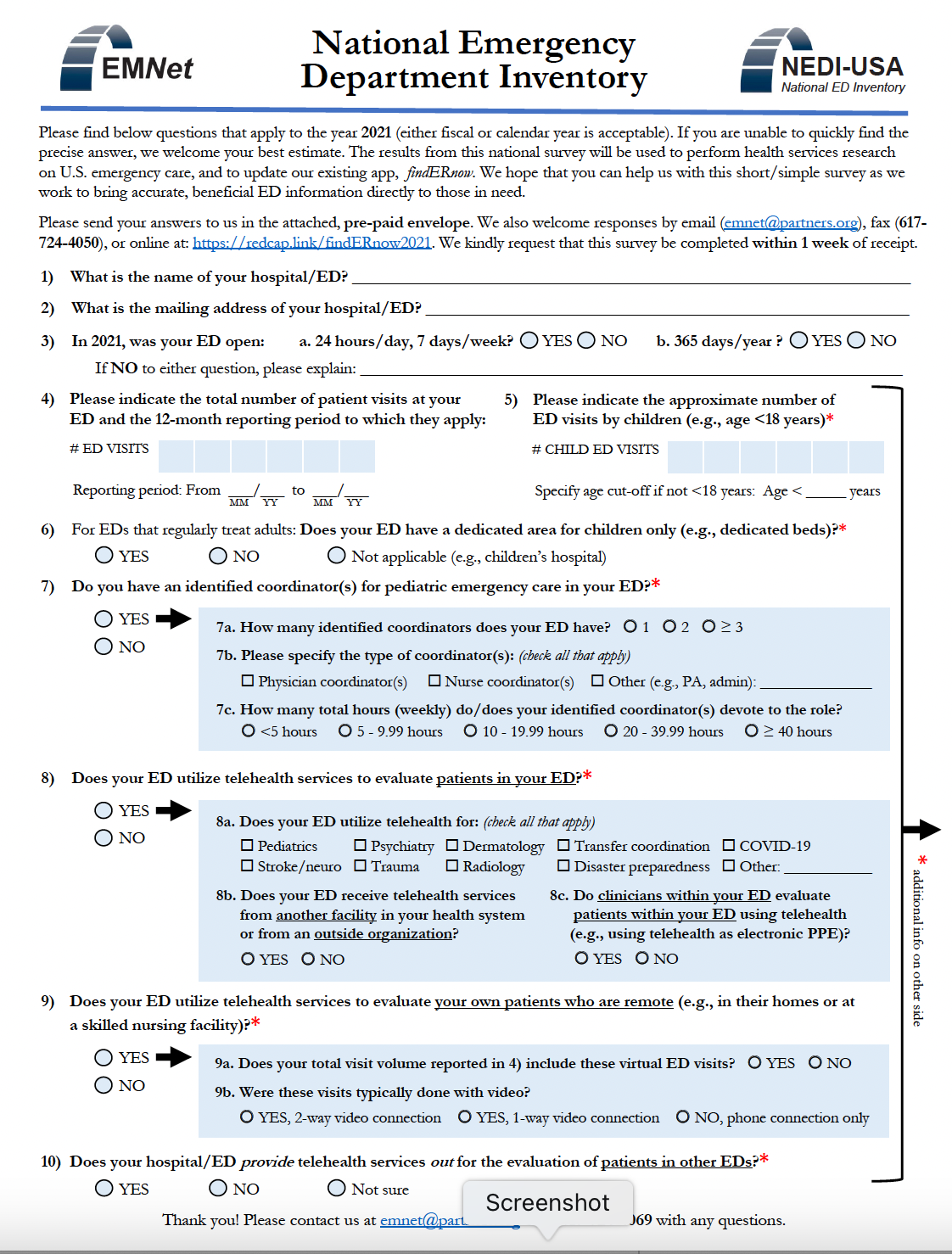


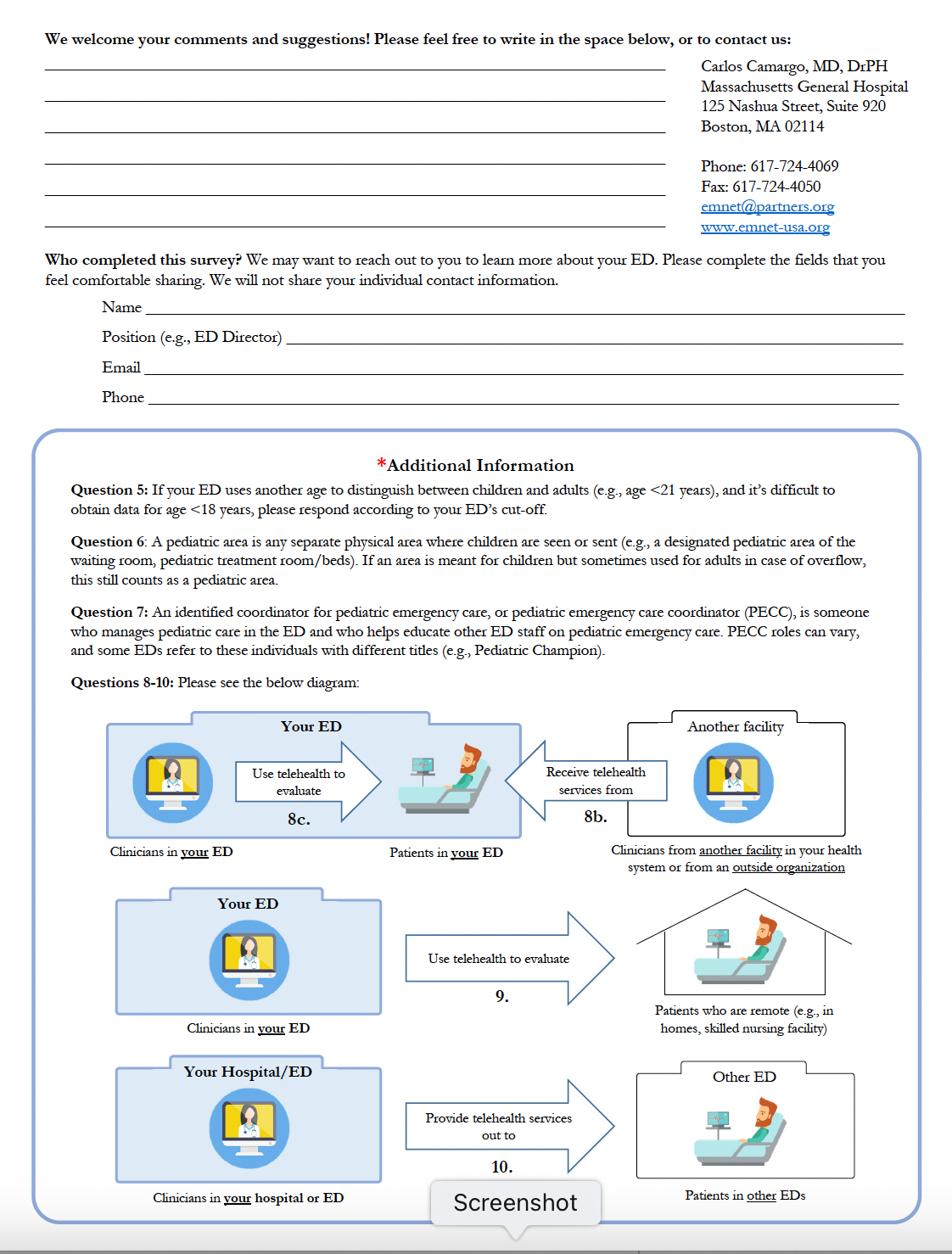


1. 2021 NEDI-California Survey


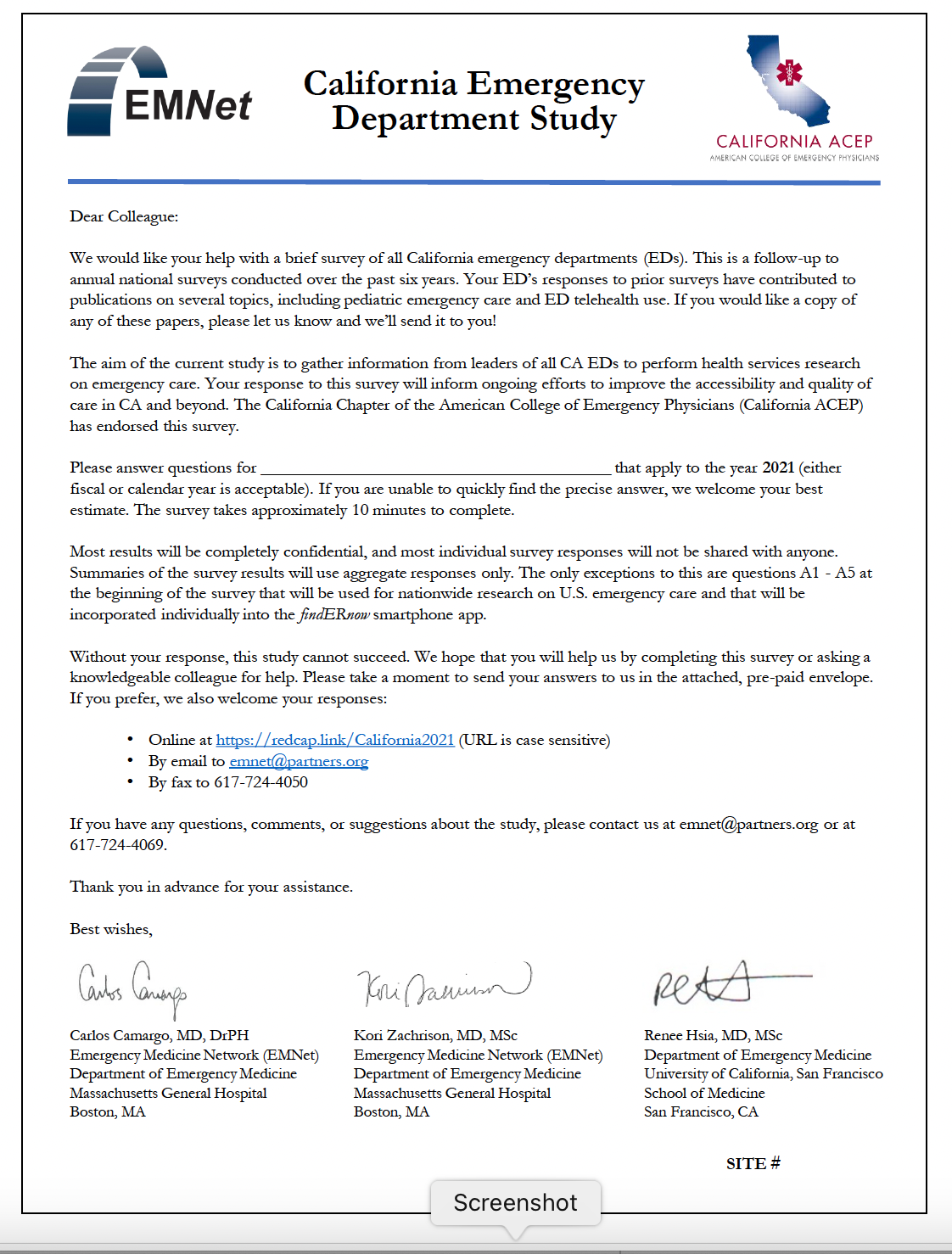


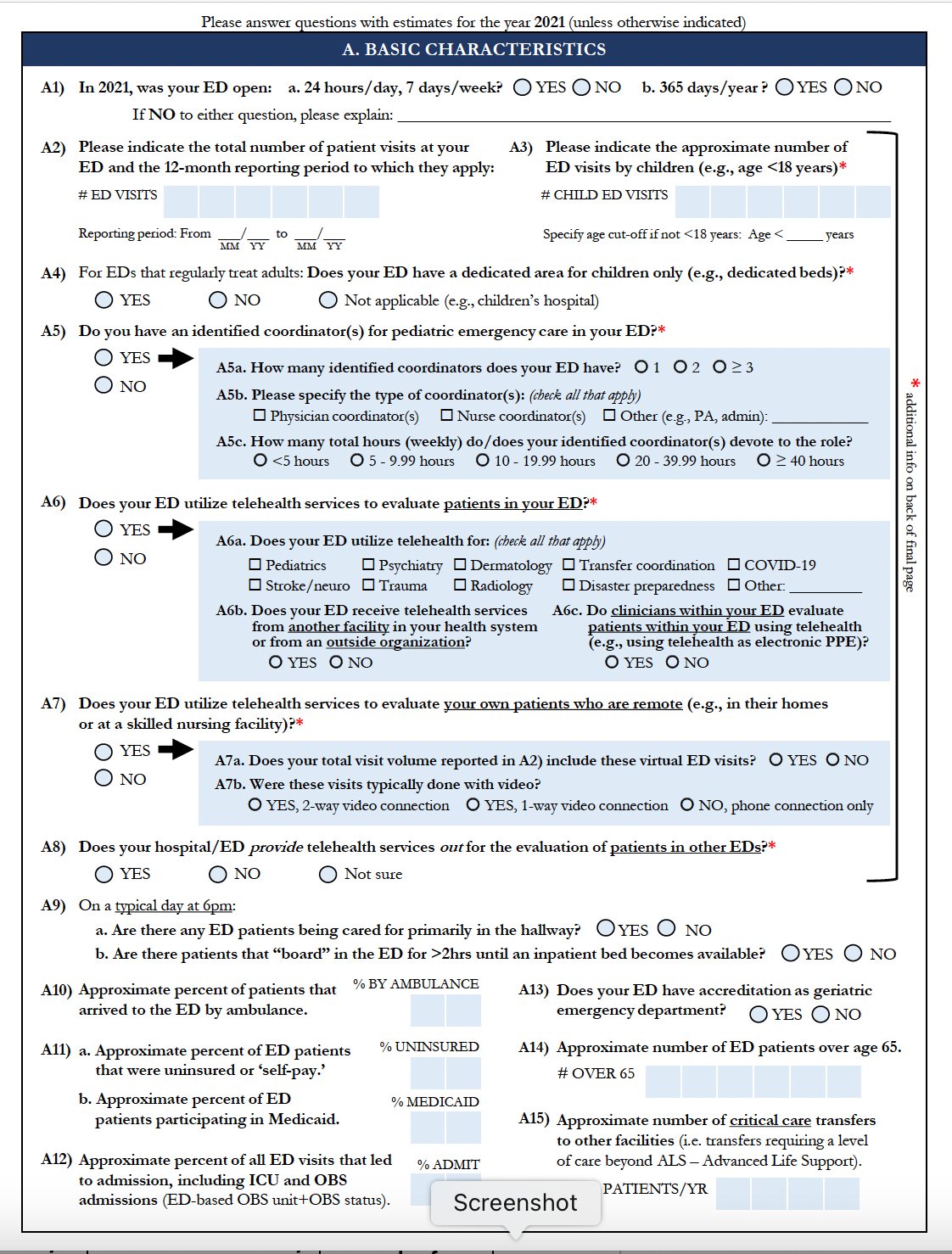


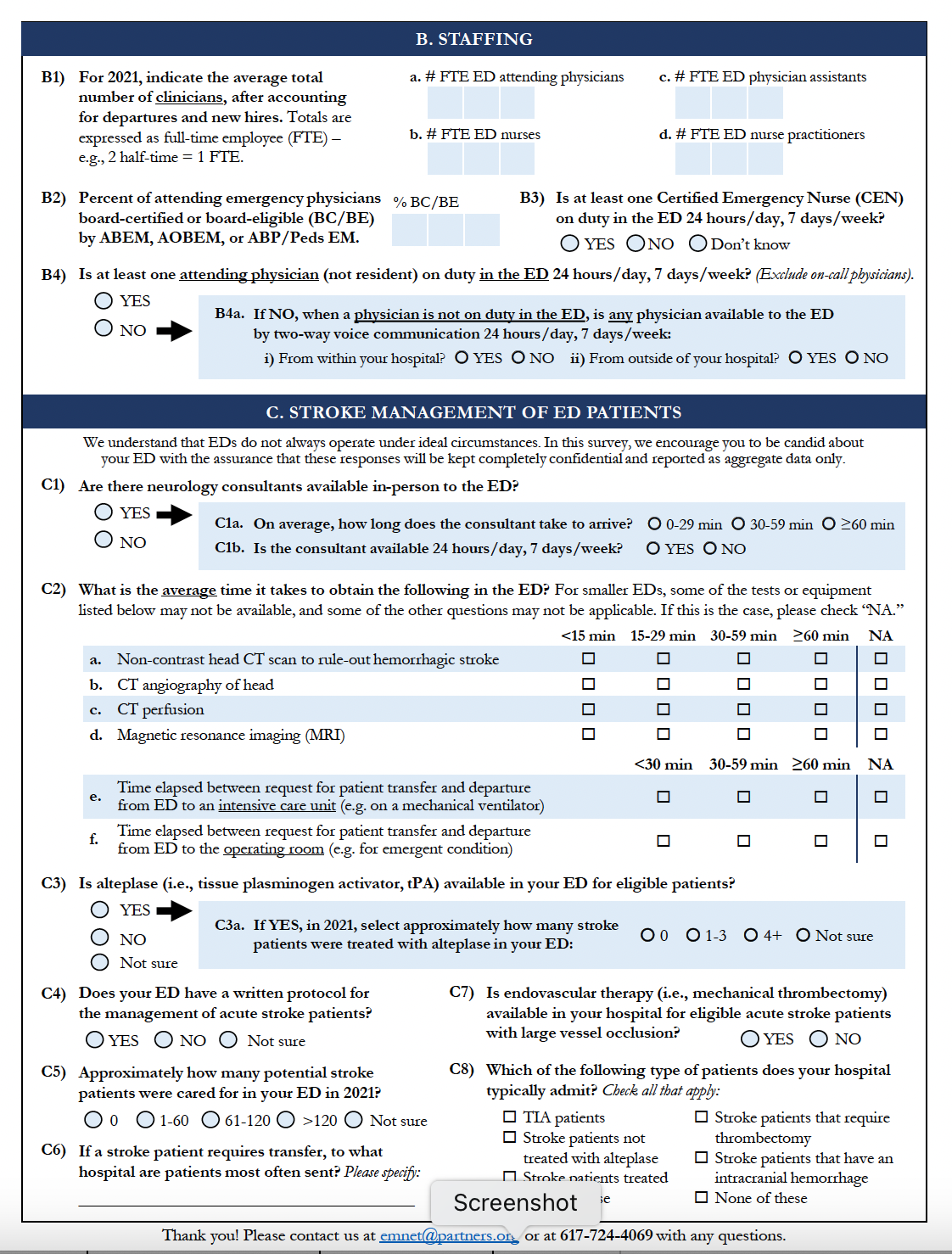


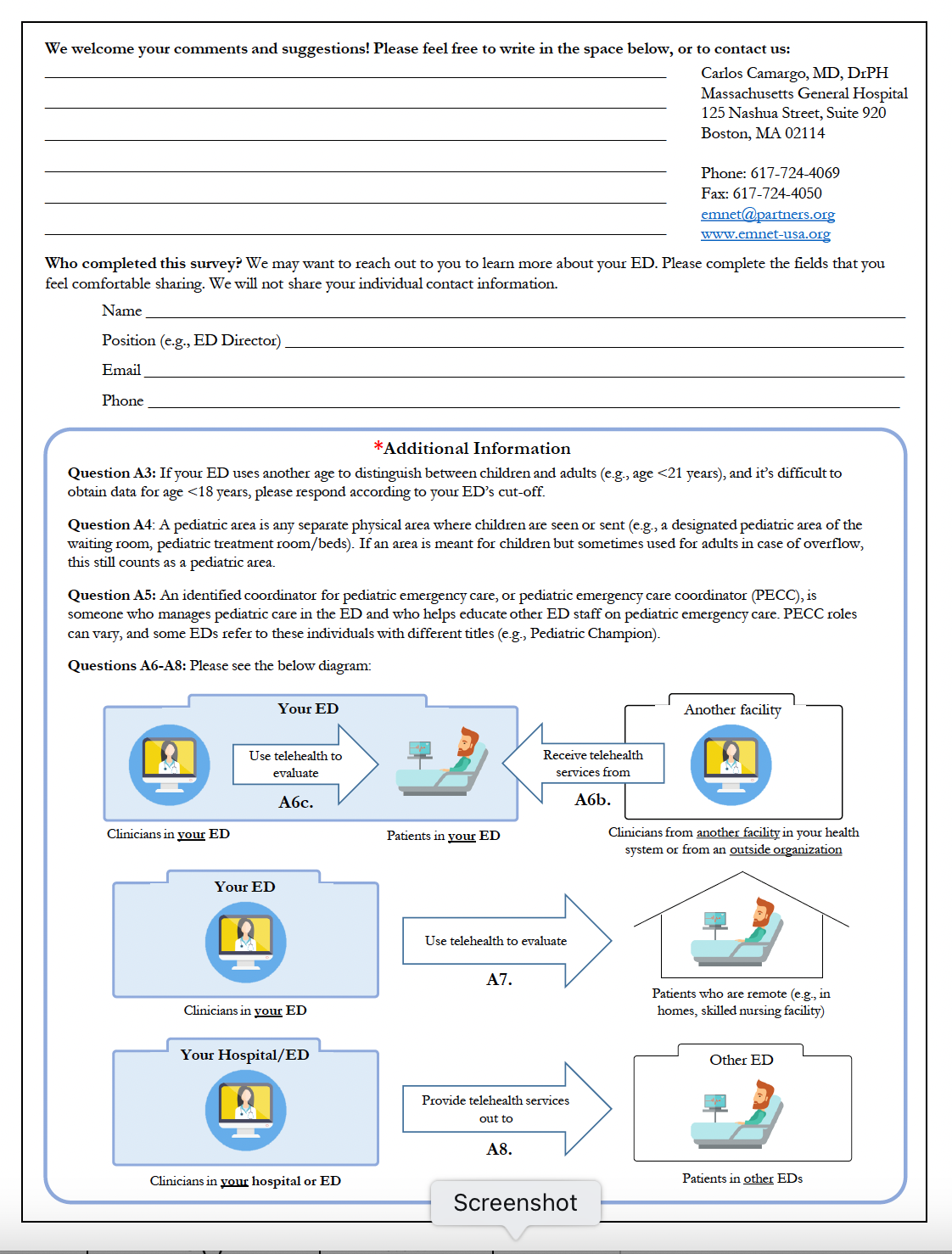
`

1. Figure. Flowchart of California Emergency Departments Included

|  |
| --- |
| 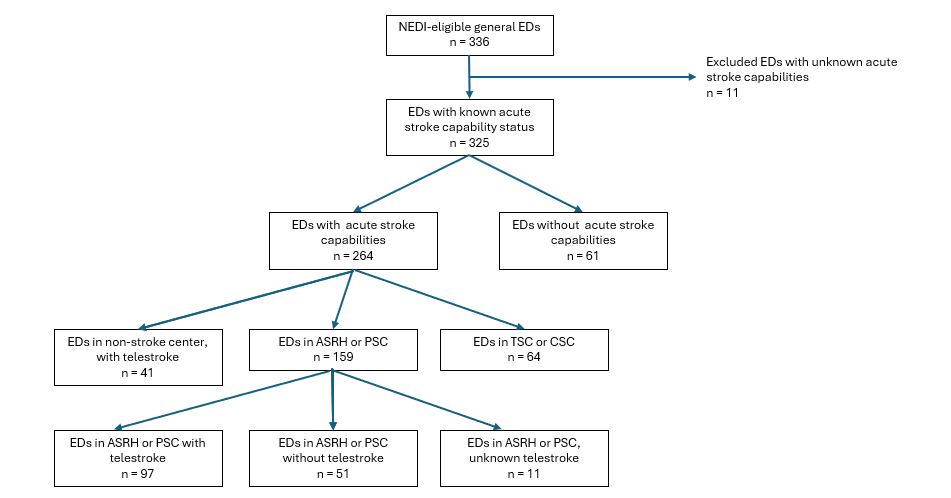 |
| **Legend**: ED emergency department; NEDI National ED Inventory; HCAI Health Care Access and Information; ASRH acute stroke ready hospital; PSC primary stroke center; TSC thrombectomy-capable stroke center; CSC comprehensive stroke center |

1. Supplemental Table 1. Relationship between Hospital Characteristics and ED Stroke Capabilities*

|  | **Unadjusted** | **Adjusted** |
| --- | --- | --- |
|  | **OR for likelihood of having stroke capabilities available (95% CI)** | **aOR for likelihood of having stroke capabilities available (95% CI)** |
| SVI | 0.33 (0.11-1.02) | 0.36 (0.09-1.42) |
| Rural ED location |  | 0.55 (0.18-1.67) |
| CAH status |  | 1.23 (0.42-3.57) |
| Academic status |  | 1.84 (0.18-19.05) |
| Annual stroke volume |  |  |
| Unknown |  | 0.10 (0.02-0.42) |
| <60 |  | 0.03 (0.01-0.10) |
| 60-120 |  | 0.22 (0.07-0.74) |
| >120 |  | Reference |

**Legend.** OR odds ratio; CI confidence interval; aOR adjusted odds ratio; SVI: social vulnerability index; ED emergency department; CAH critical access hospital

*Acute stroke capabilities defined as presence of telestroke or in an acute stroke ready hospital, primary, thrombectomy-capable, or comprehensive stroke center

1. Supplemental Table 2. Characteristics of Respondent vs. Non-Respondent EDs to the NEDI-California Survey

| **ED Characteristics** | **NEDI eligible EDs in California** | **NEDI-California survey respondents** | **NEDI-California survey non-respondents** | **p-value for difference** |
| --- | --- | --- | --- | --- |
|  | **n=325** | **n=134** | **n=191** |  |
| 2021 visit volume |  |  |  | 0.89 |
| Median, IQR | 38,031 (20,008-60,000) | 38,769 (17,153-63,213) | 38,031 (22,140-60,000) |  |
| Range | 240-164,250 | 1,095-150,000 | 240-164,250 |  |
| Rural location, n (%) | 31 (10) | 15 (11) | 16 (8) | 0.45 |
| Academic, n (%) | 21 (6) | 13 (10) | 8 (4) | 0.047 |
| CAH status, n (%) | 36 (11) | 21 (16) | 15 (8) | 0.03 |
| Social vulnerability index where ED is located, median, IQR | 1 (0-3) | 1 (0-3) | 1 (0-3) | 0.16 |

**Legend.** ED emergency department; NEDI National Emergency Department Inventory; CAH critical access hospital; IQR interquartile range
